# Bibliometric insights into systemic sclerosis with renal involvement: trends, contributions, and future directions

**DOI:** 10.1080/0886022X.2025.2463583

**Published:** 2025-02-24

**Authors:** Haochen Huang, Ling-ling Zhang, Jiaxin Zhou, Mengtao Li, Xiaofeng Zeng, Dong Xu

**Affiliations:** ^a^ Department of Rheumatology and Clinical Immunology, Peking Union Medical College Hospital, Chinese Academy of Medical Sciences, Peking Union Medical College, Beijing, China; ^b^ National Clinical Research Center for Dermatologic and Immunologic Diseases (NCRC-DID), Ministry of Science & Technology, Beijing, China; ^c^ State Key Laboratory of Complex Severe and Rare Diseases, Peking Union Medical College Hospital, Beijing, China; ^d^ Key Laboratory of Rheumatology and Clinical Immunology, Ministry of Education, Beijing, China; ^e^ Department of Rheumatology and Clinical Immunology, Beijing Shijitan Hospital, Capital Medical University, Beijing, China

**Keywords:** Systemic sclerosis, renal involvement, scleroderma renal crisis, vasculopathy, bibliometric analysis

## Abstract

**Background:**

Renal involvement is not uncommon in patients with systemic sclerosis (SSc) and presents in various forms, particularly progressing to scleroderma renal crisis (SRC), which is associated with poor prognosis. Therefore, understanding the research trends in this field is critical for advancing clinical management and therapeutic strategies.

**Methods:**

A bibliometric analysis was conducted using the Web of Science Core Collection, examining publications related to SSc and renal involvement from January 2000 to November 2024. We analyzed publication trends, key contributors, institutions, and countries.

**Results:**

A total of 1,339 publications were identified in the field of SSc and renal involvement, demonstrating an upward trend in publication volume from 2000 to 2024. These articles have been cited a total of 61,234 times, with the majority of contributions coming from the United States, Italy, and East Asian countries. The University of Michigan and University College London were particularly prominent in terms of both publication volume and collaboration networks. Keyword analysis revealed a shift in research focus, with increasing attention on clinical aspects, pathophysiological mechanisms, and vascular complications

**Conclusions:**

This study provides a comprehensive overview of the research landscape on SSc with renal involvement, highlighting the key contributors and emerging trends.

## 1. Introduction

Systemic sclerosis (SSc), also known as scleroderma, is a complex, progressive, systemic autoimmune disease characterized by widespread vascular damage and fibrosis of the skin and internal organs [1]. It typically manifests in individuals aged 30–50 years, with a female-to-male ratio of 5:1, and a global incidence of 17.6 per 100,000 people, with an annual incidence of 1.4 per 100,000 [2]. Clinically, SSc presents significant challenges to physicians due to the lack of risk factor stratification and uncertain treatment efficacy. The absence of effective therapeutic strategies contributes to a mortality rate that is 6–8 times higher than that of the general population, making SSc one of the most severe connective tissue diseases, with a substantial economic burden on both families and society [3]. The exact pathogenesis of SSc remains unclear, though it is thought to involve abnormal immune activation, fibroblast activation, and microvascular injury, leading to fibrosis and vascular remodeling in affected tissues and organs [4]. In addition to skin sclerosis, SSc frequently involves multiple organ systems, such as gastroesophageal reflux, interstitial lung disease, pulmonary hypertension, myocardial damage, and renal impairment [3]. Pathological analysis of autopsy samples has shown evidence of renal involvement in up to 80% of SSc patients [5]. Furthermore, studies suggest that up to 50% of SSc patients may experience renal involvement, which is associated with poorer prognosis. Notably, only a small subset of these patients exhibit clinical signs such as hypertension, proteinuria, or elevated serum creatinine levels, and these individuals are often also affected by other vascular complications [6,7]. Renal damage in SSc can present in various forms, including scleroderma renal crisis (SRC), ANCA-associated tubulointerstitial nephritis, and drug-induced kidney injury [8]. Among these, SRC is one of the most severe and clinically significant complications. SRC is characterized by acute hypertension and renal failure, which, if not promptly managed, can lead to patient mortality [9,10]. In recent years, advances in understanding the pathophysiology of SSc have led to the development of therapeutic strategies for renal involvement, with the use of ACE inhibitors significantly reducing the mortality associated with SRC [8,11]. Several studies have shown that the occurrence of SRC is closely associated with the presence of anti-RNA polymerase III antibodies (ARAs), which can help identify high-risk patients [12–14].

SSc with renal involvement is a rapidly evolving frontier in research, with an increasing number of studies and publications in recent years, indicating its growing prominence. Reviews on this topic have been published from various perspectives [10,15–18]. However, to date, there has been no comprehensive analysis of key authors, institutions, research progress, and emerging trends in this field. Given this gap, and leveraging our own expertise, we conducted a bibliometric analysis to systematically summarize the existing body of research. This not only provides newcomers to the field with a clear understanding of the classic issues and developments but also offers new insights and directions for future research, especially for those with long-term involvement in the field.

Bibliometrics is a research method that utilizes statistical and mathematical techniques to quantitatively and qualitatively analyze the quantity and distribution of literature [19]. Since its introduction by Pritchard in 1969, bibliometrics has become an essential tool widely applied in medicine, science, and other fields [20]. In recent years, bibliometric tools such as CiteSpace, VOSviewer, and the ‘bibliometrix’ software package have been extensively used to analyze large volumes of unstructured data, identify academic evolution in specific fields, assess research output, and predict future research trends [21–23]. To date, only three bibliometric studies on SSc have been conducted, focusing on the overall landscape of SSc, its economic implications, and pulmonary involvement [24–26]. However, to our knowledge, no bibliometric analysis has been specifically conducted in the context of SSc with renal involvement. Therefore, to fill this gap, the present study aims to objectively reveal the current state and future directions of research on SSc with renal involvement through bibliometric analysis, identifying key contributors, institutions, countries, and current research foci, while also offering insights into the evolving trends and future prospects of this field.

## 2. Methods

The relevant data for this study were retrieved and downloaded from the Web of Science Core Collection (WoSCC). The specific search strategy, inclusion, and exclusion criteria are outlined in Figure S1. For the subsequent bibliometric analysis, we employed several tools, including VOSviewer (v1.6.20), CiteSpace (v6.1 R6), the bibliometrix package (4.2.1) in R, Pajek 1.0.0.0, and Microsoft Excel 2021 for statistical analysis and visualization. Detailed methodologies can be found in the Supplementary Methods.

## 3. Results

### 3.1. Publication outputs and citation trend

A total of 1339 unique, relevant documents were identified, including 955 research articles and 384 reviews. These studies span 54 countries, 1658 institutions, 417 journals, 6800 authors, 2265 keywords, and 44,152 references, as depicted in Figure S2.

**Figure 1.**
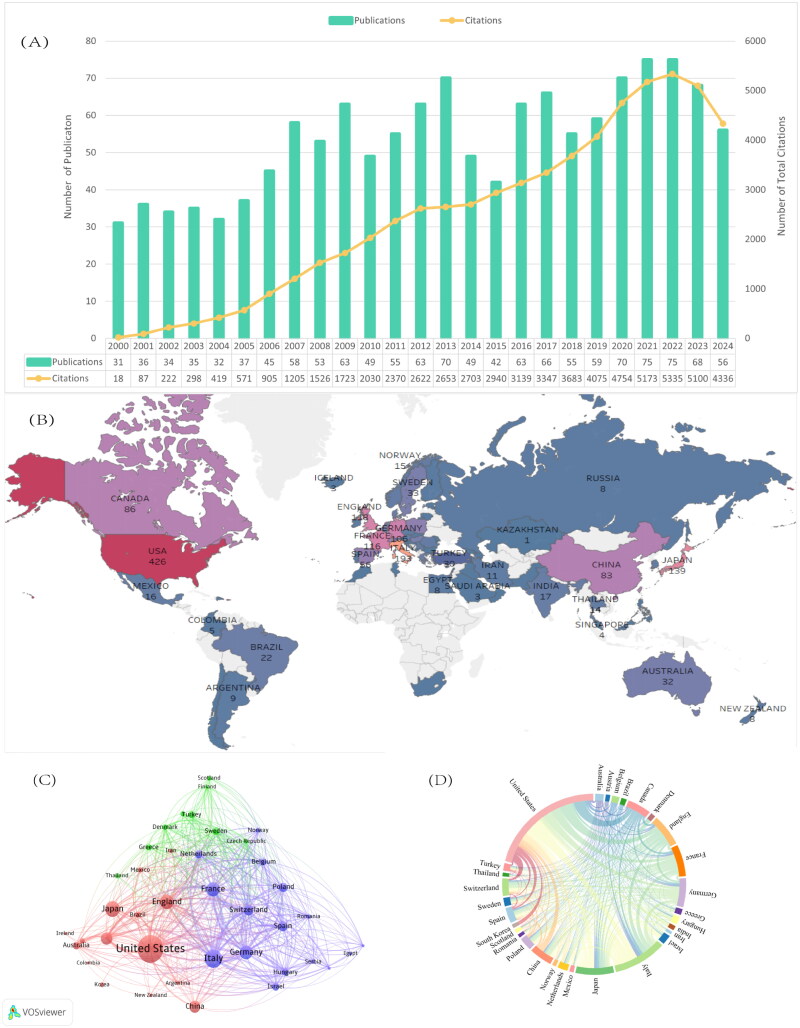
(A) Trends of annual publications and cations. (B) Visualization of global mapping of publications. (C) Collaboration mapping with a minimum number of documents of country of >5 publications based on collaboration among countries. (D) Network mapping of cooperation relationships among the main countries.

Figure 1(A) presents the annual publication trends and corresponding citation frequencies. From 2000 to 2024, the number of publications on SSc associated with renal involvement exhibited slight annual fluctuations but showed a general upward trajectory. Since data retrieval was completed in November 2024, the exact number of articles for the full year of 2024 is not captured. However, it is projected that the total for 2024 will be on par with 2023. Regarding citation metrics, these publications have been cited a total of 61,234 times, yielding an average of 45.73 citations per article. In 2022, a peak was reached with 75 papers published and 5335 citations. Additionally, as shown in Figure S3, Price’s logical growth curve indicates that the publication volume in this field is still in its early growth phase, remaining in a stage of exploration and accumulation, with an insignificant increase in output. In summary, these findings suggest that research on SSc associated with renal involvement has gained substantial attention over the past 24 years, is in a phase of rapid development, and is expected to continue growing.

### 3.2. Country/region distribution

Research in this field has been contributed by 54 countries/regions. As illustrated in Figure 1(B), the most prolific contributors are primarily from North America, Western Europe, and East Asia. Publication productivity is summarized in Table S1. The United States leads with 426 publications, followed by Italy and Japan. The U.S. stands out for its significant contribution, with the highest citation count (33,134 times) and an *H*-index of 152.

Figure 1(C) shows a global collaboration network for research on SSc associated with renal involvement, generated using VOSviewer. Only countries/regions with more than five publications were included in this analysis. Among the 40 nations meeting this criterion, the U.S., Italy, and Germany are the most connected, reflecting their prominent roles in the field. Figure 1(D) further illustrates the collaboration dynamics, with the U.S. demonstrating the most extensive partnerships, particularly with some Western European countries. These results underscore the importance of international collaboration in advancing research on SSc associated with renal involvement.

### 3.3. Institutions

As of November 2024, 1889 institutions have published articles on SSc associated with renal involvement. The University of Michigan leads with 32 publications, followed by the University of Florence and McGill University. Notably, four of the top 10 institutions are from the United States (Table S2).

**Figure 2.**
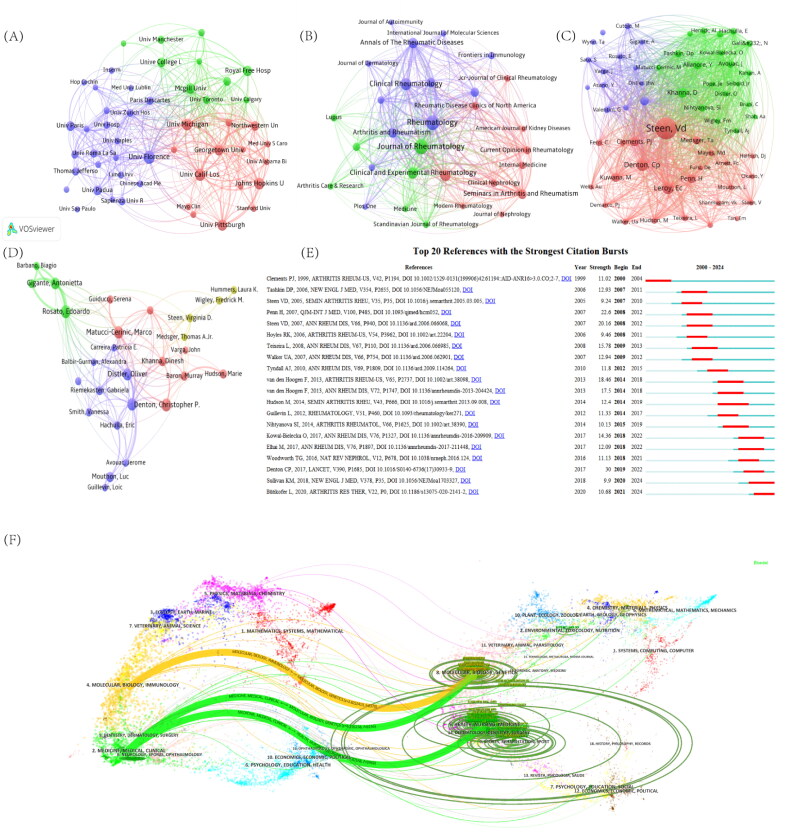
Visualization related to institutions, journals, authors, and references. (A) Network visualization showing the relationship between institutions. (B) Network visualization showing the relationship between journals. (C) Network visualization showing the co-cited relationship between authors with a minimum number of documents of author of >70 co-citations based on collaboration among authors. (D) Network visualization showing the relationship between authors. (E) The top 25 references with the strongest citation. (F) The dual-map overlay of journals. The right side of the figure is the cited literature, which is the basic research; on the right are the citing literatures, which are applied studies. The elliptic curve at the periphery shows the extent to which the influence of the cited document has spread in this field.

Centrality, used to assess the importance of nodes in a collaboration network, reveals that the University College London (UCL), despite having only 13 publications, holds the highest centrality (Centrality = 0.18) and occupies a central position in the network, reflecting its significant contribution. The collaboration network, shown in Figure 2(A), visualizes authorship for institutions with at least 10 publications, resulting in 53 institutions. These institutions form three color-coded clusters, with the University of Michigan, University of Florence, and McGill University occupying central positions in their respective clusters.

### 3.4. Journals

In the journal analysis, 417 journals have published articles on SSc with renal involvement. Table S3 shows the top 10 journals by publication count and citation frequency, respectively. Additionally, Figure 2(B) presents a visualization of the journals’ co-occurrence network. The *Journal of Rheumatology* leads with 64 articles, followed by *Rheumatology and Clinical Rheumatology* (Figure S4). Figure S5 illustrates the trend in impact factors (IFs) over the past decade for the top five journals by publication volume in this field, providing valuable insights to guide researchers in selecting appropriate journals for submission. Cited journals are those frequently referenced by other publications, reflecting their impact in the field. Despite publishing only 28 articles, *Arthritis & Rheumatology* has a much higher citation frequency than other journals, underscoring its significant academic influence. It is worth noting that *The New England Journal of Medicine* and *The Lancet* are among the top international academic journals in SCI in various fields.

The dual-map overlay of journals reveals citation patterns between journals. As shown in Figure 2(F), the orange path indicates that studies in molecular, biological, and genetic journals are often cited by research in molecular, biological, and immunological journals, while the green path shows that research in medical and clinical journals frequently cites studies from molecular, biological, genetic, nursing, and healthcare journals.

### 3.5. Authors and coauthors

A total of 6800 authors from different countries contributed to this research, with the distribution of their publication output generally following Lotka’s law (Figure S6). Table S4 lists the top 10 most prolific authors and the top 10 most co-cited authors in studies related to co-occurrence. The most published authors are Professor Christopher P. Denton from UCL in the UK and Professor Edoardo Rosato from the University of Palermo in Italy, both having published 28 articles. They are followed by Marco Matucci-Cerinic (25 publications) and Yannick Allanore (25 publications). Co-cited authors refer to those who are cited together in one or more publications. The top three most co-cited authors are Virginia D. Steen (1367 citations), Professor Christopher P. Denton (406 citations), and Edward C. Leroy (399 citations). These scholars have made indispensable contributions to the knowledge base of SSc with renal involvement.

Figure 2(D) shows the author collaboration network, with three distinct research clusters. Denton, Allanore, and Steen are central to their respective clusters. Co-citation analysis via VOSviewer identified 58 authors with at least 70 citations, with Steen, Denton, and Dinesh Khanna having the most connections in the network (Figure 2(C)).

### 3.6. Co-cited references and references burst

Table S5 lists the top 10 most-cited articles in this field. The study by Leroy et al. published in the *Journal of Rheumatology* in 1988, stands out with an impressive 227 co-citations, making it the most cited article. All top 10 articles have been cited more than 80 times, highlighting their significant impact. Among these influential and seminal papers, terms such as ‘scleroderma renal crisis’ and ‘classification of systemic sclerosis’ are frequently mentioned. Of these studies, six focus on SRC, while three address the classification of SSc subtypes.

Citation bursts refer to the phenomenon where the frequency of citations for a particular article experiences a sudden increase over a short period. Using CiteSpace V (version 6.1 R6), we conducted a citation burst analysis and identified a total of 20 references exhibiting citation bursts (Figure 2(E)). The onset of citation bursts can be traced back to 2000, with the most recent citation burst occurring in 2021, which is still ongoing. Among the references, the study by Denton et al. published in 2017, had the highest burst value (strength = 30).

These references provide valuable insights for novice researchers into the classic literature and current state of research on SSc associated with renal involvement, offering direction for advancing breakthroughs in this field.

### 3.7. Cluster and evolution of keywords

#### 3.7.1. Keyword co-occurrence analysis and heat map

**Figure 3.**
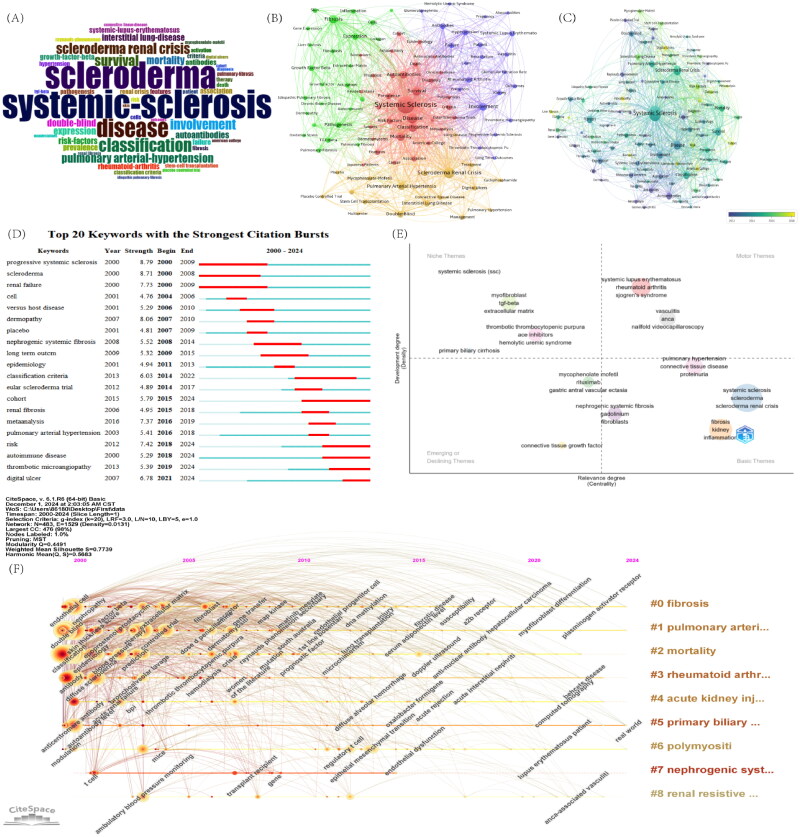
The analysis of keywords. (A) Word cloud plot of keywords. (B) The network visualization map of keywords. (C) Keyword overlay visualization. (D) CiteSpace visualization map of the top 20 keywords with the strongest citation bursts. (E) The thematic map produced by bibliometrix. (F) Visualization of the timeline for keywords spanning from 2000 to 2024.

Using VOSviewer, a total of 2265 keywords were identified in this field. Table S6 lists the top 50 keywords after removing duplicates and merging synonyms. Excluding disease-related terms, the top five keywords are SRC, Classification Criteria, Disease, Survival, and Fibrosis. Figure 3(A) visualizes these keywords in a word cloud.

**Figure 4.**
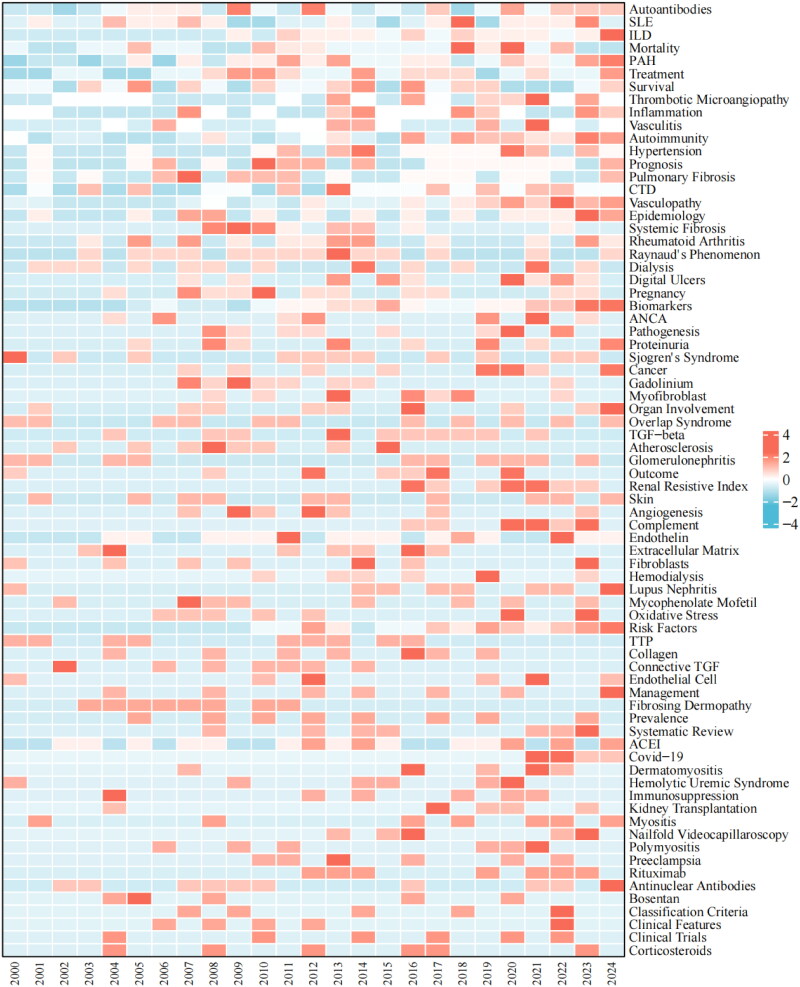
The heat map of keywords.

As shown in Figure 3(B), a co-occurrence analysis of keywords identified 108 high-frequency terms, each occurring more than 20 times. Using the clustering feature, four distinct clusters were generated: the red cluster, with keywords such as ‘Risk factors,’ ‘Survival,’ ‘Mortality,’ and ‘Diagnosis,’ is strongly associated with clinical research and clinical data; the green cluster, including ‘Growth factor-β,’ ‘Fibrosis,’ ‘Collagen,’ and ‘Gene Expression,’ relates to pathogenesis research and bioinformatics; the purple and yellow clusters are associated with vascular involvement and complications, featuring keywords such as ‘Scleroderma renal crisis,’ ‘Interstitial lung disease,’ ‘Thrombotic microangiopathy,’ and ‘Digital ulcers.’ Figure 3(C) displays the keyword evolution over time, showing shifts in focus. Purple nodes represent keywords from earlier periods, while yellow nodes indicate emerging hot topics. Initially, terms like ‘Antinuclear antibody,’ ‘Renal failure,’ ‘Raynaud’s phenomenon,’ and ‘Placebo’ were central. However, recent years have seen increasing attention on terms like ‘Digital ulcers,’ ‘Liver fibrosis,’ ‘Vasculopathy,’ ‘Biomarker,’ ‘Cohort,’ and ‘Meta-analysis,’ suggesting these may become key research areas in the future. This shift reflects a transition in the field from classic diagnostic methods and common manifestations to the exploration of complex pathogenesis and high-evidence clinical research. The CiteSpace timeline and heat map (Figures 3(F) and 4) visually depicts the evolution of these keywords, revealing significant changes in their frequency and reflecting the shift in research priorities. In the heatmap, keywords such as ‘Biomarker,’ ‘Risk Factors,’ and ‘Vasculopathy’ have shown a significant increase in frequency in recent years. Furthermore, since 2015, there has been a notable rise in scientific exploration of mechanisms such as autoantibodies, myofibroblast differentiation, and endothelial dysfunction. These keywords likely represent current research hotspots related to SSc with renal involvement.

#### 3.7.2. Keyword burst analysis and thematic map

Figure 3(D) lists the top 20 keywords with the strongest citation bursts. ‘Digital ulcer,’ ‘Thrombotic microangiopathy,’ and ‘Autoimmune disease’ are among the newest keywords, indicating a shift in focus toward mechanisms related to microvascular involvement. This trend is expected to persist through 2024 and likely continue in the future.

The thematic map provides insights into the classification and development trends of research topic keywords, reflecting both their development (density) and relevance (centrality) (Figure 3(E)). The upper-right quadrant, containing ‘systemic lupus erythematosus,’ ‘Sjögren’s syndrome,’ ‘rheumatoid arthritis,’ and ‘vasculitis,’ represents motor themes, which are highly developed core topics driving research in this field. These themes are closely related to other systemic diseases, indicating that renal involvement in multiple autoimmune disorders is currently a major academic focus. The lower-right quadrant, labeled as basic themes, contains core topics like ‘scleroderma renal crisis,’ ‘fibrosis,’ and ‘pulmonary hypertension.’ These represent foundational concepts that underpin more specialized research. The upper-left quadrant, known as niche themes, highlights emerging but more specialized research topics, such as ‘TGF-β,’ ‘myofibroblasts,’ and ‘extracellular matrix,’ which are related to the pathogenesis of SSc with renal involvement. This suggests that these mechanisms are poised to lead innovation in the field and are crucial to its continued development.

## 4. Discussion

This study employs data from the Web of Science (WoS Core Collection, SCI-Expanded) and bibliometric methods to comprehensively review and describe the development of scientific publications on the topic of SSc with renal involvement.

Due to missing keyword fields in literature prior to 2000, we focused on publications related to SSc with renal involvement from 1 January 2000 to 26 November 2024. We explored the research output patterns, international collaboration, journal influence, and academic contributions by countries, regions, and authors. A total of 54 countries, 1658 institutions, and 6800 researchers contributed to these studies. Over the past 24 years, the number of publications in this field has fluctuated, peaking in 2013 with 70 papers before decreasing sharply, only returning to this level in 2020. A significant decline in publications occurred between 2014 and 2015, though citation counts continued to rise, indicating the presence of influential publications that gained wide academic consensus during this period. Based on our analysis, we hypothesize that a key review published by Steen in 2014 in *Presse Medicale* titled ‘Kidney involvement in systemic sclerosis’ might have contributed to this surge, along with another review by Woodworth et al. in 2016 in *Nature Reviews Nephrology* titled ‘Scleroderma renal crisis and renal involvement in systemic sclerosis’ [16,17]. This trend continued with the 2023 publication by Scheen et al. in *Autoimmunity Reviews* titled ‘Renal involvement in systemic sclerosis,’ which updated the latest knowledge in the field [9]. These reviews, which cover topics such as definitions, pathogenesis, pathology, epidemiology, predictive factors, and clinical applications, have significantly contributed to the understanding of SSc with renal involvement and serve as valuable references for researchers. Furthermore, they provide an excellent resource for newcomers to rapidly grasp the historical development of the field. Finally, in predicting future publication volumes, the results from Price’s logical growth curve suggest that the field is currently experiencing slow growth, with relatively limited scientific output and innovation. The curve displays a very gradual exponential increase, with output showing only modest growth, and it is expected to enter a phase of rapid expansion around 2030. Overall, the growing academic interest in SSc with renal involvement is evident from both the number of annual publications and citation counts.

By analyzing the distribution of research across 54 countries and 1658 institutions, we observed an imbalance in the global research on SSc with renal involvement. The increase in publications is largely driven by a few countries, particularly the United States, which contributes the most, followed by Italy and Japan. Notably, four of the top 10 most productive institutions are based in the United States. Interestingly, China is the only developing country among the top 10 nations. However, none of the top 10 institutions belong to China. Citation counts serve as a significant indicator of the impact of published work in this field, providing valuable insights for further scientific inquiry. The total citation count for the United States is 33,134, 3.37 times higher than Italy and 8.57 times higher than Japan. Additionally, the United States leads in both average citation count and *H*-index, underscoring its dominant influence in advancing research in this area. International collaboration is particularly frequent among countries such as the United States, Italy, France, and England. In 2004, clinical experts and researchers from European countries established the European Scleroderma Trials and Research Group (EUSTAR), which has significantly advanced scientific progress in the field of SSc through multinational cooperation and data sharing [27,28]. While China and other developing countries have relatively limited international collaboration, recent years have seen increased research investment, leading institutions and scholars in these countries to enhance their focus across various fields, making substantial contributions to scientific advancement. However, many developing nations face challenges in detecting renal involvement due to limitations in technology, resulting in insufficient clinical data for patients. In China, for instance, routine testing for ARAs is not commonly available, although some regions are able to send samples to specialized laboratories for analysis. Furthermore, the lack of standardized quality control has raised concerns regarding the accuracy of these diagnostic methods. Given the significance of these antibodies in SRC, the absence of such data undermines the credibility of many studies in this area. As awareness of this critical biomarker grows, the implementation of routine ARA testing has become an urgent priority. To enhance the research output and quality of developing countries, we believe that strengthening research infrastructure is essential, including improving laboratory facilities, data storage, and computing platforms, as well as promoting academic resource sharing. Additionally, developing countries should focus on local priorities and address regional issues while fostering international collaboration and engagement, particularly by joining global research networks, which can elevate academic output and increase international visibility. Finally, it is crucial to promote open-access publishing to enhance the exposure and citation of publications, thereby increasing the impact of research findings. In conclusion, enhanced international collaboration could help bridge academic barriers, fostering a positive feedback loop to promote high-quality research.

A diverse array of prestigious journals provides high-quality literature resources for researchers seeking cutting-edge knowledge in this field. A total of 417 journals have published related articles, with the top 10 journals contributing 25.84% of the total publications. The *Journal of Rheumatology* leads in publication volume, followed by other high-impact journals such as *Arthritis & Rheumatology*, *Annals of the Rheumatic Diseases*, and *Autoimmunity Reviews*. This underscores the increasing attention the field has received from researchers in autoimmune diseases, and reflects the willingness of these journals to accept research related to the topic. Further co-citation analysis using VOSviewer reveals the significant influence of these journals. Notably, prominent journals like *The New England Journal of Medicine* and *The Lancet* also appear in the co-citation analysis, indicating their broad interdisciplinary impact. Additionally, nine out of the 10 most co-cited references are published in these high-impact journals. These journals span multiple disciplines, including rheumatology, immunology, molecular biology, biochemistry, clinical research methodologies, and statistics, demonstrating the widespread recognition of this field in both basic and clinical research. Citing articles from such reputable journals can also enhance the credibility of future publications. These findings provide valuable insights for researchers when submitting manuscripts and referencing literature.

The analysis of authors reveals that the most prolific and most frequently cited researchers in this field are C.P. Denton from UCL and V.D. Steen from Georgetown University School of Medicine. Notably, although Khanna Dinesh from the University of Michigan has only published 16 papers, his total citation count is an impressive 2276, with a high co-citation frequency as well. These statistics highlight the significant influence of these authors, whose scholarly publications and research are likely to represent cutting-edge topics or emerging trends in the field, making them highly relevant for further study. C.P. Denton and his team have been conducting research on the pathogenesis of SSc and large European cohorts associated with SSc since the 1990s, validating the efficacy of various drugs in treating SSc patients. They have also been instrumental in writing and revising treatment recommendations for SSc, as proposed by the European League Against Rheumatism (EULAR) [29–31]. In the early stages of their research on renal involvement, Denton et al. focused on the diagnostic value of various autoantibodies across different organs, as well as the application of ACE inhibitors in patients with renal involvement, exploring the role of ARAs and anti-fibrinogen antibodies (AFAs) in diagnosing SSc-related renal involvement [32,33]. Although the use of ACE inhibitors significantly reduces the mortality associated with SRC, the use of ACE inhibitors before SRC occurs does not improve long-term outcomes in patients with SRC, Denton’s team reports, so its prophylactic use is not supported [34–37]. More recently, Denton’s team identified an increase in the expression of candidate proteins GPATCH2L and CTNND2 in the kidney tissues of ARA-positive patients, suggesting these may play a potential role in the onset of SRC [13]. Additionally, the damage-associated molecular pattern protein S100A4 has been identified as a promising therapeutic target in SSc, showing a definitive fibrogenic effect and a correlation with SRC [38].

Based on the co-occurrence analysis of keywords and the top 20 keywords with the strongest citation bursts, two relatively prominent research trends have emerged after assessing the current status. The most significant trend pertains to the study of SSc with renal involvement, particularly in relation to vasculopathy. Keywords associated with vascular complications, such as pulmonary arterial hypertension, digital ulcers, and thrombotic microangiopathy, have appeared as focal points in Figure 3(B,C), with citation bursts still ongoing in 2024 [39–41]. It is widely accepted that immune dysregulation, vascular damage, and fibrosis are the three central mechanisms driving the pathogenesis of SSc [1]. While much of the existing research focuses on the mechanisms of fibrosis formation in the later stages of the disease, there has been comparatively limited investigation into the vascular alterations occurring early in SSc pathogenesis. In fact, nearly, all SSc patients exhibit varying degrees of vascular damage. Vascular lesions are not only a key pathological event that triggers clinical symptoms related to SSc and vascular structural changes, but they also serve as the link between inflammation and fibrosis in the disease mechanism [42,43]. The pathogenesis of renal vascular involvement remains poorly understood. Existing literature suggests that endothelial cells may play a crucial role in microvascular injury. The initial vascular damage in SSc-related renal vasculopathy may be triggered by environmental factors, autoimmune attack by anti-endothelial cell antibodies (AECAs), or γδT cells, leading to endothelial cell activation. This activation upregulates the expression of adhesion molecules such as ICAM-1, GlyCAM-1, E-selectin, and P-selectin, which subsequently recruits more inflammatory cells, including Th2, TH17 cells, mast cells, and macrophages, into the vasculature and surrounding tissues [44–47]. Activated endothelial cells can also differentiate into myofibroblasts through the pathological process of endothelial-to-mesenchymal transition (EndoMT), producing extracellular matrix components that contribute to intimal proliferation, lumen narrowing, vascular occlusion, and tissue fibrosis. Additionally, endothelin-1 (ET-1) has been reported to promote the progression of this process [48]. Alterations in circulating vasoactive factors can also contribute to vascular damage. SSc patients often exhibit elevated levels of VEGF and its receptors, and studies have shown that excessive VEGF expression leads to endothelial cell hyperproliferation and abnormal vascular growth, resulting in disorganized vasculature and chaotic blood vessel structures [49].

Another research hotspot focuses on identifying the risk factors associated with renal involvement in SSc and predicting disease onset. Among all forms of renal involvement in SSc, SRC is the most severe complication and a major cause of death in SSc patients [7,9]. Although several risk factors for SRC have been proposed, including myocardial involvement, anemia, high-dose corticosteroids (>15 mg/day), and diffuse SSc, most of these remain challenging to apply clinically [33,50–53]. Myocardial involvement reduces cardiac output, activates the renin–angiotensin axis, and triggers the onset of SRC. Early studies also suggested that renal dysfunction lead to severe sodium and water retention, further exacerbating systemic hypertension and potentially worsening cardiac contractility [54]. Anemia results in insufficient oxygen delivery to tissues and organs, leading to renal ischemia and exacerbating kidney damage. Additionally, anemia increases blood viscosity, which may reduce blood flow to the glomeruli [55]. The use of high-dose corticosteroids has a permissive effect on catecholamine responses, further decreasing renal blood flow and negatively impacting glomerular filtration rate, thereby increasing renal burden [56]. In recent years, the rise of machine learning has enabled many research teams to use high-risk disease factors as predictive variables [57]. Various machine learning algorithms are employed to construct clinical prediction models that can efficiently forecast disease outcomes, enhance understanding of key disease determinants, and provide more accurate assessments of disease progression and prognosis. In the context of SSc with renal involvement, similar studies have been reported by Mu and coworkers [58], but due to the limited sample sizes, these models still require further validation before they can be applied in clinical practice.

## 5. Limitation

While this paper provides valuable insights into the global research trends and interest in SSc with renal involvement, there are notable limitations that warrant careful consideration. First, our analysis was limited to English-language articles indexed in the WOS SCI-Expanded database, meaning that studies from other databases, such as PubMed and Embase, as well as non-English publications, were not included. This could potentially overlook important literature in the field. Second, recently published high-quality papers may not yet have accumulated sufficient citations to be adequately recognized in our analysis. However, bibliometric analysis is inherently time-sensitive and requires continuous updates to provide a more comprehensive understanding of the current research landscape and future trends. Third, due to the extensive time span of the literature collection and the varying recording methods employed by different publishers, a significant number of fields related to research funding models are missing, preventing us from conducting a thorough and reliable analysis in this area. Lastly, variations in institutional access to the WOS database may lead to slight discrepancies in the number of articles retrieved using the same search query, although this does not significantly affect the overall results and trends.

## 6. Conclusions

This bibliometric analysis explores research trends on SSc with renal involvement from 2000 to 2024. It reveals a rising publication trajectory, with major contributions from the United States, Italy, and Japan. The study identifies key research areas, including clinical mechanisms and vascular complications. Citation analysis and collaboration networks highlight the field’s growing influence, yet emphasize the need for stronger international cooperation, especially in developing countries like China. These insights offer valuable direction for future research and clinical advancements in the SSc community.

## Supplementary Material

AA1.txt

Supplementary_method_and_materials new.docx

## Data Availability

Data will be made available on request.
