# Supplementary material for: Bibliometric insights into systemic sclerosis with renal involvement: trends, contributions, and future directions
Source: Ren Fail. 2025 Feb 24;47(1):2463583. doi: 10.1080/0886022X.2025.2463583 (PMC11864008; doi:10.1080/0886022X.2025.2463583)
Supplement: Supplementary_method_and_materials new.docx [file IRNF_A_2463583_SM2277.docx]

Supplementary Method

2.1 Data sources

The literature search for this study was conducted using the Science Citation Index Expanded (SCI-Expanded) within the Web of Science Core Collection (WoSCC), a comprehensive and widely recognized database hosted by Clarivate Analytics. WoSCC is considered the gold standard for bibliometric research due to its rigorous indexing of high-quality, peer-reviewed scholarly journals across a broad range of disciplines(1). Numerous studies have demonstrated that Web of Science excels in accurate document classification compared to other databases, such as PubMed and Scopus, making it a preferred tool for bibliometric analyses. Its extensive repository of scholarly resources provides a robust foundation for conducting in-depth, high-fidelity bibliometric assessments(2, 3).

2.2 Retrieval methods and literature screening

This study focused on articles published between January 1, 2000, and November 26, 2024, to complement the current research landscape. All retrieval and download tasks were completed within a single day to minimize potential biases due to daily database updates. The search query used was as follows:(TS=("systemic sclerosis" OR "sclerosis, systemic" OR "scleroderma") AND TS=("kidney" OR "nephron" OR "renal" OR "nephropathy" OR "kidney disease" OR "renal disease" OR "disease, kidney" OR "disease, renal" OR "kidney pathology" OR "renal pathology" OR "renal disorder" OR "kidney disorder" OR "renal injury" OR "kidney injury" OR "renal impairment" OR "kidney impairment" OR "kidney failure" OR "renal failure" OR "renal crisis")).

Inclusion criteria were as follows: (i) Articles published between 2000 and 2024; (ii) Publication language: English; (iii) Publication type: article or review. Exclusion criteria included: (i) Non-English publications; (ii) Publication type: meeting abstracts, editorials, letters, proceedings, early access articles, book chapters, corrections, or news items; (iii) Duplicates. The primary data retrieval was independently conducted by two researchers, and any discrepancies were resolved through discussion with a third researcher. Ultimately, 1,339 articles were included in the analysis. All relevant references were exported in TXT format as "full records and references." Further details regarding the screening process are provided in Figure S1.

Additionally, this study adhered to the principles outlined in the Declaration of Helsinki(4). Since the scope of this research excluded animal and human clinical trials, no ethical concerns were involved.

2.3 Bibliometric analysis

In this study, we employed a variety of software tools to perform a comprehensive bibliometric analysis.

VOSviewer, developed by van Eck et al., is a software tool that integrates clustering algorithms to assist researchers in constructing and visualizing co-occurrence networks that extract key information from scientific literature(5). In addition, CiteSpace is a software tool developed by Chaomei Chen for visualizing and analyzing trends in scientific literature. It uses bibliometric analysis and network visualization to identify key publications, authors, and emerging topics, helping researchers map the intellectual structure and evolution of a research field(6).

Microsoft Office Excel 2021 was used to conduct quantitative analysis of publication counts and total citation numbers. VOSviewer (v1.6.20) and other tools were utilized to analyze countries, institutions, journals, co-cited journals, authors, co-cited authors, and keyword co-occurrence. These analyses facilitated the construction of collaboration networks, co-citation networks, and co-occurrence networks. Visual representations of these networks were provided through maps generated by VOSviewer and other software. Additionally, the R package "bibliometrix" (v4.2.1) was employed to analyze thematic evolution and generate keyword cloud maps.

1. Pranckutė R. Web of Science (WoS) and Scopus: The titans of bibliographic information in today’s academic world. Publications. 2021;9(1):12.

2. Noruzi A, Gholampour B, Gholampour S, Kister J. Scientific transition from chemistry to information and communication sciences: A tribute to Professor Henri Dou, a pioneer of competitive intelligence in France. Informology. 2023;2(1).

3. Gholampour B, Gholampour S, Noruzi A. Research trend analysis of information science in France based on total, cited and uncited publications: A scientometric and altmetric analysis. Informology. 2022;1(1):7-26.

4. Goodyear MD, Krleza-Jeric K, Lemmens T. The declaration of Helsinki. British Medical Journal Publishing Group; 2007. p. 624-5.

5. Van Eck NJ, Waltman L. Citation-based clustering of publications using CitNetExplorer and VOSviewer. Scientometrics. 2017;111:1053-70.

6. Synnestvedt MB, Chen C, Holmes JH, editors. CiteSpace II: visualization and knowledge discovery in bibliographic databases. AMIA annual symposium proceedings; 2005: American Medical Informatics Association.

Supplementary Materials


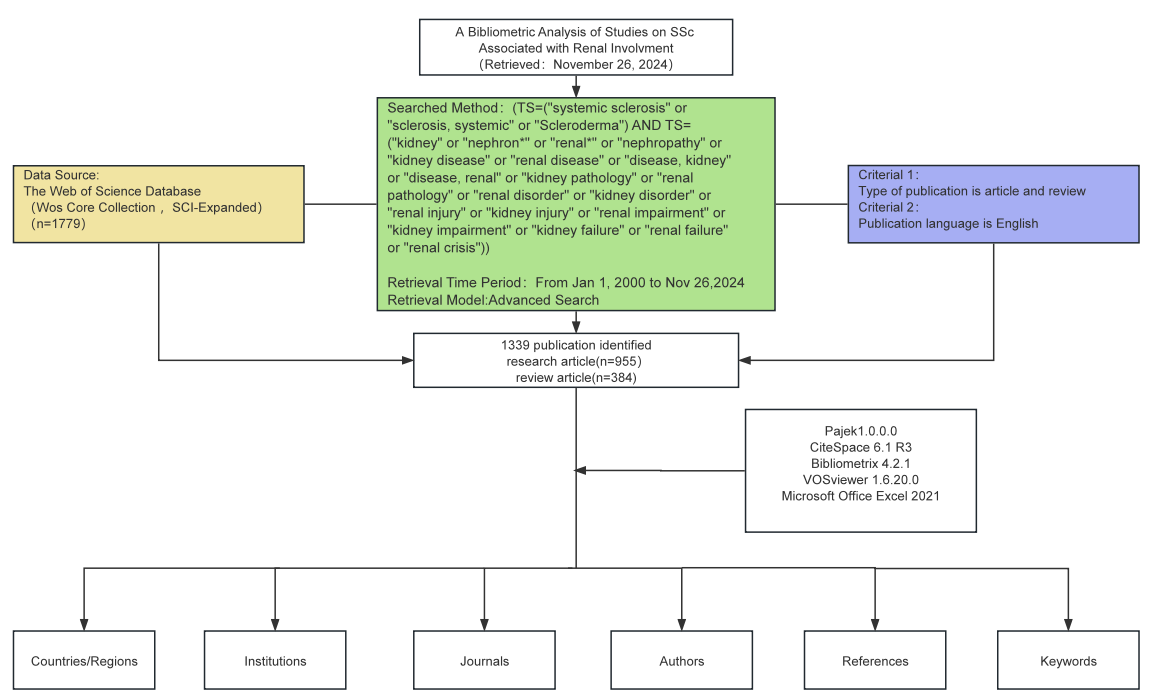


**Supplementary Figure 1:Flowchart of selected publications for this study**


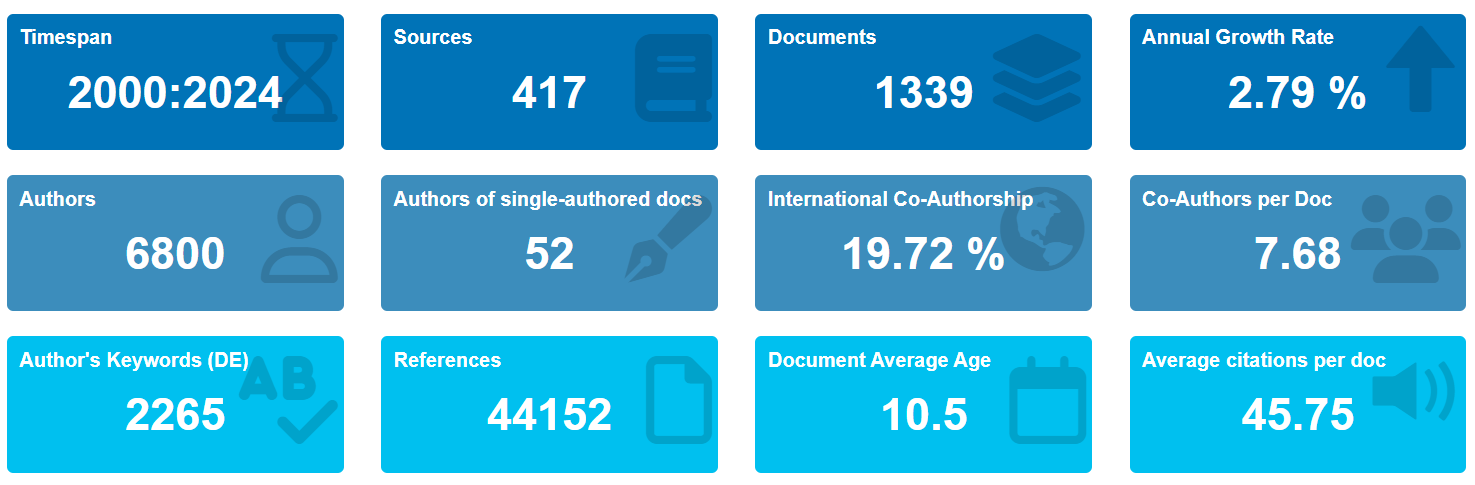


**Supplementary Figure 2:The main information of the included documents.**


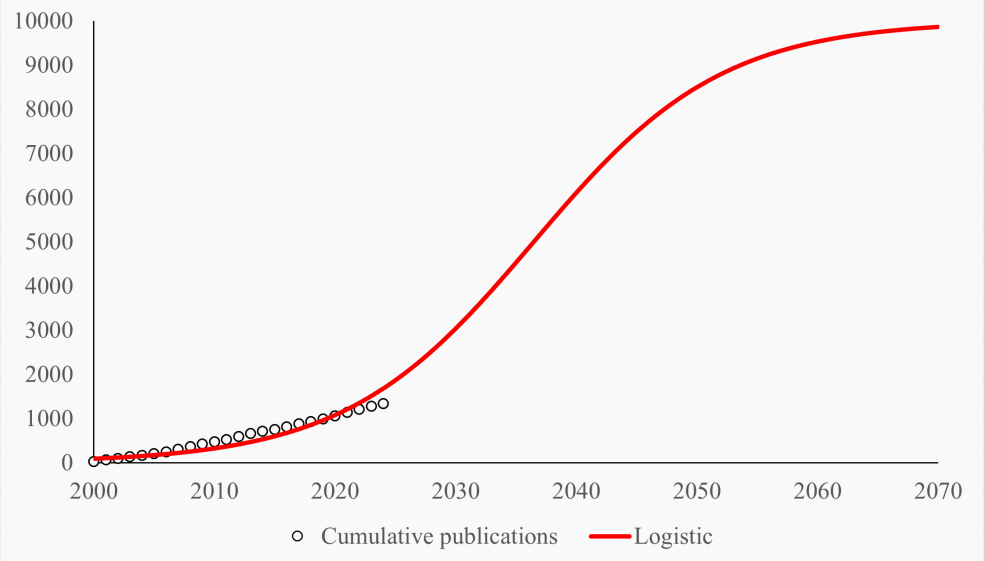


**Supplementary Figure 3:The result of Price's Logistic Growth Curve**


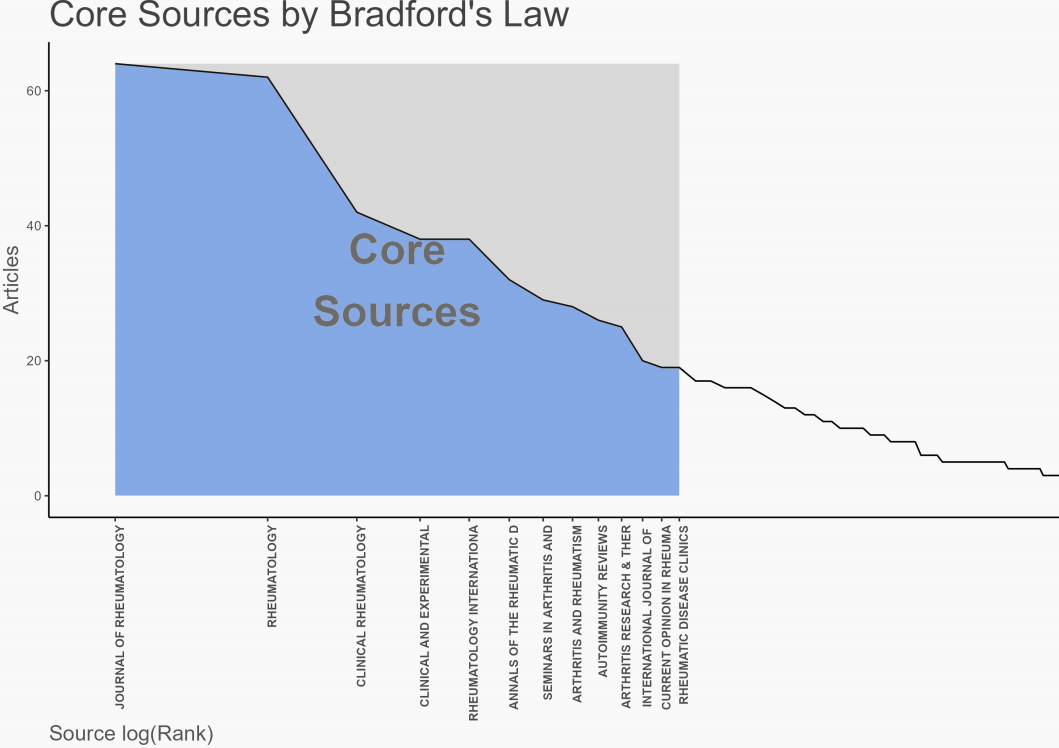


**Supplementary Figure 4: Core journals determined according to Bradford’ s law.**

**
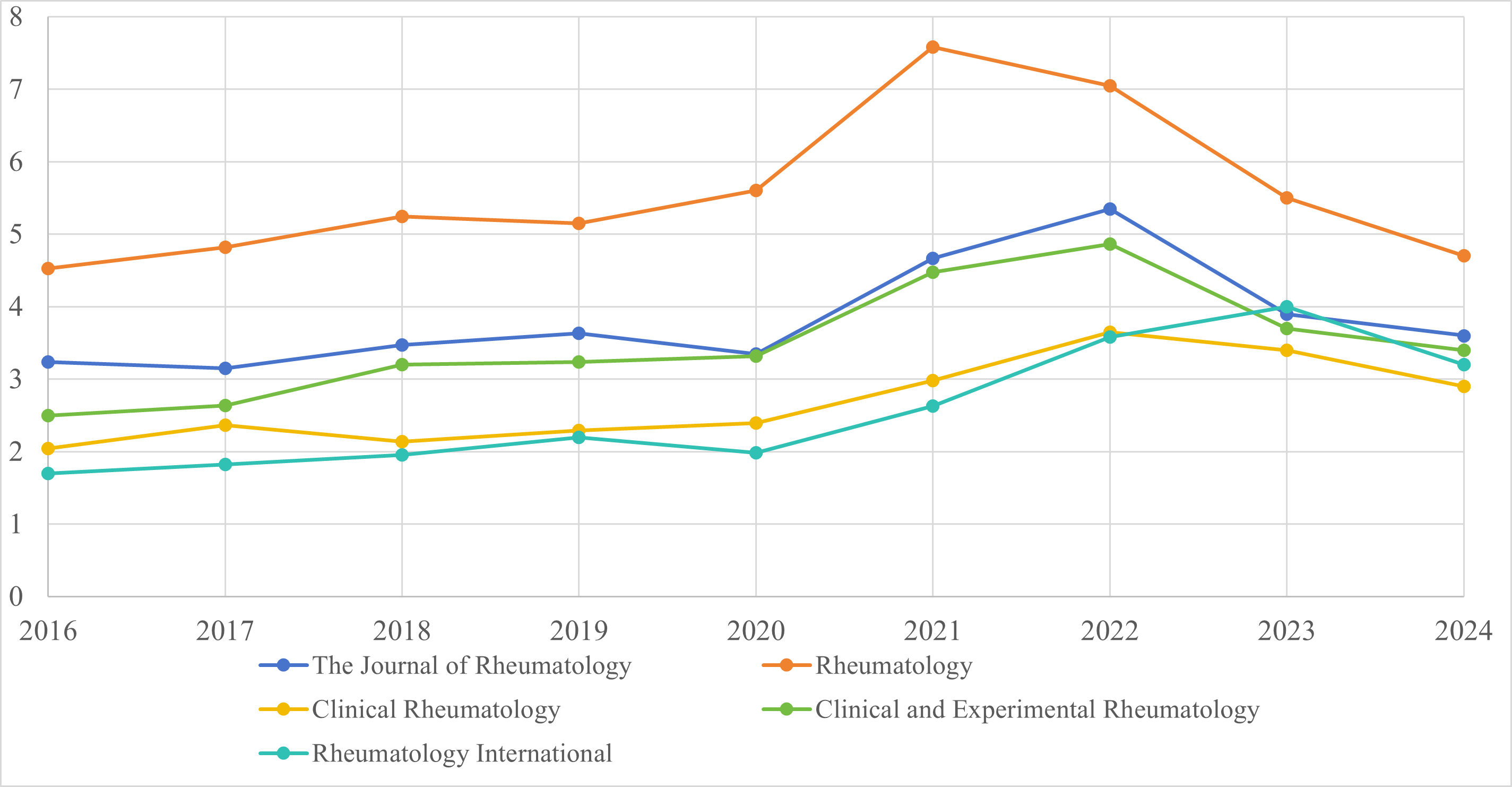
**

**Supplementary Figure 5:The trends of Top 5 journals’IF in** [**SSc associated renal involvement**](https://pubmed.ncbi.nlm.nih.gov/38319374/) **research.**


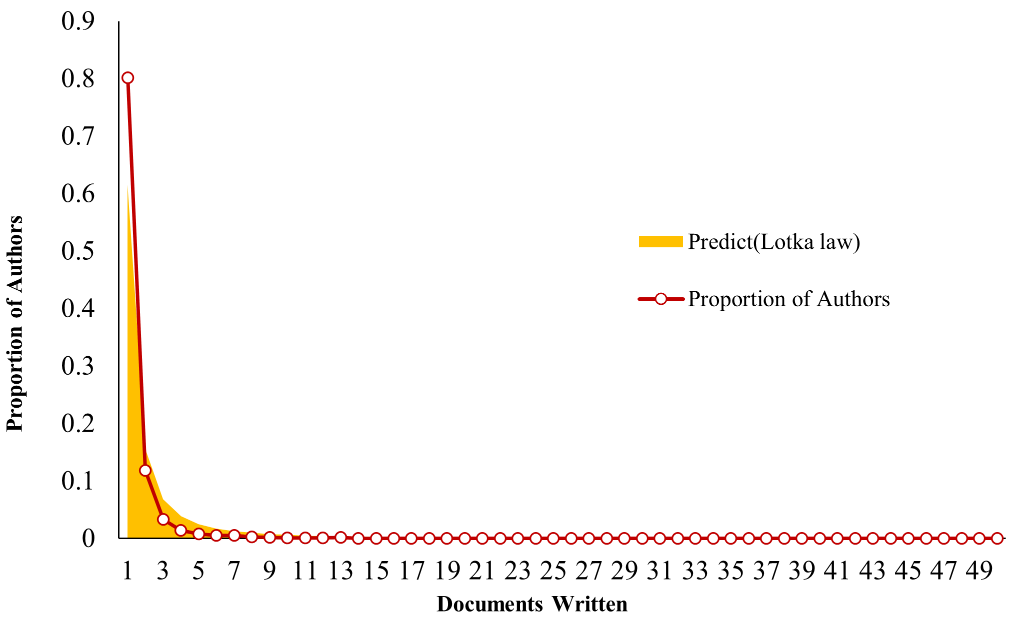


**Supplementary Figure 6:The result of Lotka’ law.**

| Rank | Country/Region | Publications | Total citations | Average citations | H-index |
| --- | --- | --- | --- | --- | --- |
| 1 | United States | 426 | 33134 | 77.78 | 152 |
| 2 | Italy | 193 | 9823 | 50.90 | 83 |
| 3 | Japan | 139 | 3862 | 27.78 | 41 |
| 4 | England | 118 | 8711 | 73.82 | 78 |
| 5 | France | 116 | 7762 | 66.91 | 94 |
| 6 | Germany | 106 | 5981 | 56.42 | 74 |
| 7 | Canada | 86 | 5406 | 62.86 | 58 |
| 8 | China | 83 | 2306 | 27.78 | 42 |
| 9 | Switzerland | 64 | 5403 | 84.42 | 46 |
| 10 | Spain | 56 | 2543 | 45.41 | 61 |

**Supplementary Table 1: Top 10 countries/regions in** [**SSc associated renal involvement**](https://pubmed.ncbi.nlm.nih.gov/38319374/) **research.**

| Rank | Institution | Country | Publication | Total citation | Centrality |
| --- | --- | --- | --- | --- | --- |
| 1 | University of Michigan | United States | 32 | 4210 | 0.07 |
| 2 | University of Florence | Italy | 28 | 3310 | 0.1 |
| 3 | McGill University | Canada | 17 | 1085 | 0.02 |
| 4 | Johns Hopkins University | United States | 17 | 2025 | 0.14 |
| 5 | Northwestern University | United States | 16 | 2161 | 0.01 |
| 6 | Université Paris Descartes | France | 14 | 1022 | 0.05 |
| 7 | Sapienza University of Rome | Italy | 14 | 168 | 0.01 |
| 8 | University of California, Los Angeles | United States | 13 | 2849 | 0.04 |
| 9 | University College London | England | 13 | 2621 | 0.18 |
| 10 | University of Toronto | Canada | 12 | 2078 | 0.04 |

**Supplementary Table 2: Top 10 institutions in** [**SSc associated renal involvement**](https://pubmed.ncbi.nlm.nih.gov/38319374/) **research.**

| Rank | Journal | Count | Citation | IF | JCR | Co-cited Journal | Citation | IF | JCR |
| --- | --- | --- | --- | --- | --- | --- | --- | --- | --- |
|  |  |  |  | 2023 | 2023 |  |  | 2023 | 2023 |
| 1 | The Journal of Rheumatology | 64 | 2995 | 3.6 | Q2 | Arthritis & Rheumatology | 3350 | 11.4 | Q1 |
| 2 | Rheumatology | 62 | 2467 | 4.7 | Q1 | Annals of the Rheumatic Diseases | 3162 | 20.3 | Q1 |
| 3 | Clinical Rheumatology | 42 | 809 | 2.9 | Q2 | The Journal of Rheumatology | 3102 | 3.6 | Q2 |
| 4 | Clinical and Experimental Rheumatology | 38 | 694 | 3.4 | Q2 | Rheumatology | 2033 | 4.7 | Q1 |
| 5 | Rheumatology International | 38 | 555 | 3.2 | Q2 | The New England Journal of Medicine | 1258 | 96.2 | Q1 |
| 6 | Annals of the Rheumatic Diseases | 32 | 4757 | 20.3 | Q1 | Clinical and Experimental Rheumatology | 1000 | 3.4 | Q2 |
| 7 | Seminars in Arthritis and Rheumatism | 29 | 1875 | 4.6 | Q1 | Annals of Internal Medicine | 831 | 19.6 | Q1 |
| 8 | Arthritis & Rheumatology | 28 | 3844 | 11.4 | Q1 | Seminars in Arthritis and Rheumatism | 821 | 4.6 | Q1 |
| 9 | Autoimmunity Reviews | 26 | 1559 | 9.2 | Q1 | Journal of Biological Chemistry | 815 | 4.0 | Q2 |
| 10 | Arthritis Research & Therapy | 25 | 1060 | 4.4 | Q1 | Lancet | 809 | 98.4 | Q1 |

**Supplementary Table 3:The top 10 journals ,co-cited journals related to renal involvement in SSc**

| Rank | Author | Count | Citation | Average citation | Co-cited Author | Citation |
| --- | --- | --- | --- | --- | --- | --- |
| 1 | Denton, Christopher P. | 28 | 2814 | 100.5 | Steen, Vd | 1367 |
| 2 | Rosato, Edoardo | 28 | 435 | 15.54 | Denton, Cp | 406 |
| 3 | Matucci-Cerinic, Marco | 25 | 1628 | 65.12 | Leroy, Ec | 399 |
| 4 | Allanore, Yannick | 25 | 1224 | 48.96 | Khanna, D | 295 |
| 5 | Distler, Oliver | 24 | 990 | 41.25 | Clements, Pj | 237 |
| 6 | Gigante, Antonietta | 23 | 304 | 13.22 | Kuwana, M | 226 |
| 7 | Mouthon, Luc | 18 | 804 | 44.67 | Van Den Hoogen, F | 215 |
| 8 | Khanna, Dinesh | 16 | 2276 | 142.25 | Penn, H | 210 |
| 9 | Baron, Murray | 15 | 939 | 62.6 | Allanore, Y | 199 |
| 10 | Hudson, Marie | 15 | 571 | 38.07 | Medsger, Ta | 184 |

**Supplementary Table 4:The top 10 authors ,co-cited authors related to renal involvement in SSc**

| Title | Co-citation | Year | First author | Journal |
| --- | --- | --- | --- | --- |
| Preliminary criteria for the classification of systemic sclerosis (scleroderma) | 201 | 1980 | Alfonse, T | Arthritis & Rheumatology |
| Scleroderma (systemic sclerosis): classification, subsets and pathogenesis | 277 | 1988 | LeRoy, EC | The Journal of Rheumatology |
| Outcome of renal crisis in systemic sclerosis: relation to availability of angiotensin converting enzyme (ACE) inhibitors | 134 | 1990 | Steen, Vd | Annals of Internal Medicine |
| Case‐control study of corticosteroids and other drugs that either precipitate or protect from the development of scleroderma renal crisis | 134 | 1998 | Steen, Vd | Arthritis & Rheumatology |
| Long-term outcomes of scleroderma renal crisis | 110 | 2000 | Steen, Vd | Annals of Internal Medicine |
| Predictors and outcomes of scleroderma renal crisis: the high‐dose versus low‐dose d‐penicillamine in early diffuse systemic sclerosis trial | 85 | 2002 | DeMarco, PJ | Arthritis & Rheumatology |
| Scleroderma renal crisis: patient characteristics and long-term outcomes. | 143 | 2007 | Penn, H | QJM：Monthly Journal of the Association of Physicians |
| Changes in causes of death in systemic sclerosis, 1972–2002 | 138 | 2007 | Steen, Vd | Annals of the Rheumatic Diseases |
| Clinical risk assessment of organ manifestations in systemic sclerosis: a report from the EULAR Scleroderma Trials And Research group database | 95 | 2007 | Walker, UA | Annals of the Rheumatic Diseases |
| Defective vasculogenesis in Systemic Sclerosis is related to apoptotic phenotype in bone marrow endothelial progenitors | 90 | 2008 | Gianelli, U | Annals of the Rheumatic Diseases |

**Supplementary Table 5:The top 10 co-cited references with the most citations.**

| Rank | Keywords | Counts | Rank | Keywords | Counts |
| --- | --- | --- | --- | --- | --- |
| 1 | Systemic Sclerosis（SSc） | 1252 | 26 | Cells | 44 |
| 2 | Scleroderma Renal Crisis（SRC） | 283 | 27 | Death | 44 |
| 3 | Classification Criteria | 188 | 28 | Digital Ulcers | 44 |
| 4 | Disease | 185 | 29 | Kidney | 44 |
| 5 | Survival | 135 | 30 | Risk | 44 |
| 6 | Fibrosis | 130 | 31 | Therapy | 44 |
| 7 | Pulmonary Arterial Hypertension（PAH） | 127 | 32 | Patient | 43 |
| 8 | Mortality | 109 | 33 | Skin | 42 |
| 9 | Involvement | 107 | 34 | Stem-Cell Transplantation | 42 |
| 10 | Interstitial Lung Disease（ILD） | 106 | 35 | Activation | 41 |
| 11 | Autoantibodies | 103 | 36 | Diagnosis | 40 |
| 12 | Growth Factor-Beta(GF-β) | 102 | 37 | Inflammation | 40 |
| 13 | Systemic Lupus Erythematosus（SLE） | 99 | 38 | Vasculitis | 40 |
| 14 | Double-Blind | 77 | 39 | Raynauds Phenomenon | 39 |
| 15 | Expression | 77 | 40 | Epidemiology | 38 |
| 16 | Prevalence | 69 | 41 | Pulmonary Fibrosis | 38 |
| 17 | Risk Factors | 67 | 42 | Renal Fibrosis | 38 |
| 18 | Hypertension | 66 | 43 | Outcomes | 37 |
| 19 | Antibodies | 63 | 44 | Management | 36 |
| 20 | Pathogenesis | 60 | 45 | Connective Tissue Disease（CTD） | 34 |
| 21 | Rheumatoid Arthritis（RA） | 60 | 46 | Mycophenolate-Mofetil | 34 |
| 22 | Association | 55 | 47 | American College | 33 |
| 23 | Failure | 55 | 48 | Treatment | 33 |
| 24 | Features | 49 | 49 | Cancer | 32 |
| 25 | Criteria | 48 | 50 | Placebo Controlled Trial | 32 |

**Supplementary Table 6:The top 50 keywords related to renal involvement in SSc**
